# Mechanisms and Therapeutic Strategies for Endocrine Resistance in Breast Cancer: A Comprehensive Review and Meta-Analysis

**DOI:** 10.3390/cancers17101653

**Published:** 2025-05-14

**Authors:** Asiya Khan, Sandeep Sisodiya, Mehreen Aftab, Pranay Tanwar, Showket Hussain, Vivek Gupta

**Affiliations:** 1 Multidisciplinary Research Unit, Government Institute of Medical Sciences, Greater Noida 201310, India; ak.asiyakhan1@gmail.com; 2 Department of Pathology, Government Institute of Medical Sciences, Greater Noida 201310, India; 3 Cellular and Molecular Diagnostics (Molecular Biology Group), ICMR—National Institute of Cancer Prevention and Research, Ministry of Health and Family Welfare, Government of India, Noida 201301, India; sandeepsisodiya99@gmail.com (S.S.); mehreen_aftab2004@yahoo.com (M.A.); 4 Symbiosis School of Biological Sciences (SSBS), Symbiosis International (Deemed University) (SIU), Pune 412115, India; 5 Lab Oncology Unit, Dr. B.R.A. Institute Rotary Cancer Hospital, All India Institute of Medical Sciences, New Delhi 110029, India; pranaytanwar@gmail.com

**Keywords:** breast cancer, endocrine resistance, immunotherapy, targeted therapy

## Abstract

**Background:** As per recent scenarios, drug resistance is a significant challenge in treating breast cancer for several reasons, such as genetic mutations, altered signaling pathways, and tumor microenvironment. Endocrine resistance is one of the biggest significant barriers to treatment, particularly in hormone receptor-positive (HR+) breast cancers, which depends on estrogen or progesterone signaling for growth. While therapies such as tamoxifen, aromatase inhibitors, and selective estrogen receptor degraders (SERDs) have effectively targeted these pathways, many patients develop resistance, rendering them less effective over time, which is driving a need for innovative therapeutics to treat breast cancer and overcome drug resistance and better treatment outcomes. Recent studies suggest that combining the different therapies, including immunotherapy, targeted therapy, chemotherapy, etc., with endocrine therapy, may bypass the endocrine resistance. **Methodology**: We conducted a comprehensive systematic review and meta-analysis examining the molecular mechanisms of endocrine resistance and evaluating randomized clinical trial outcomes, overall survival and progression-free survival in endocrine-resistant breast cancer patients treated with endocrine therapy, targeted therapy, immunotherapy, or chemotherapy. **Results**: We have analyzed 35 randomized clinical trial studies for different therapies along with combination therapy, and our results demonstrated that supplementary or additional therapies in endocrine resistance breast cancer patients have better progression-free and overall survival. **Conclusions**: The current study has demonstrated that combination therapies may have good survival results and patient outcomes in endocrine resistance. Also, This review sheds light on current challenges in drug resistance and the future direction of cancer treatment through a comprehensive analysis of these emerging treatment approaches to improve patient outcomes.

## Simple Summary

In the current scenario, drug resistance is a significant obstacle for treating breast cancer, especially in hormone receptor-positive patients depending on estrogen or progesterone. Although several drugs, such as aromatase inhibitors and tamoxifen, have been successful in the treatment of breast cancer, many patients acquire resistance, therefore, compromising the efficacy of the treatments. The current study investigates the biological causes of endocrine resistance and assesses clinical trial results to find better therapeutic management. We evaluated the advantages of combining endocrine therapy with targeted therapy, immunotherapy, and chemotherapy and included data from 35 randomized clinical trials. Our results imply that combination treatments may increase patient survival and disease progression. This study offers insightful analysis of novel strategies to improve patient outcomes and therapy for breast cancer.

## 1. Introduction

Breast cancer remains the most common cancer in women globally [1]. GLOBOCAN 2022 data indicates that breast cancer is the most prevalent cancer among women globally in terms of both incidence and fatality rates. It constituted around 2.3 million new cases worldwide, representing a substantial fraction of total cancer diagnoses. Breast cancer results in elevated death rates, mainly attributable to delayed diagnosis and restricted access to treatment and drug resistance [2,3].

Endocrine therapy is fundamental in the treatment of hormone receptor-positive (HR+) breast cancer, representing around 70% of all breast cancer cases. Therapies like selective estrogen receptor modulators (SERMs), aromatase inhibitors (AIs), and selective estrogen receptor degraders (SERDs) have markedly enhanced survival outcomes by targeting estrogen signaling, a pivotal factor in tumor proliferation in HR+ breast cancer [4]. Despite the initial effectiveness of these treatments, the development of endocrine resistance—either de novo or acquired—remains a significant clinical challenge. Over 30% of metastatic HR+ breast cancer patients develop endocrine resistance, leading to disease progression; the underlying mechanisms are complex and involve ER alterations, cross-talk with growth pathways (PI3K/AKT/mTOR, CDK4/6), and changes in the tumor microenvironment. Additionally, mutations in PIK3CA (encoding the catalytic subunit of PI3K) and loss of PTEN (a tumor suppressor that negatively regulates PI3K signaling) lead to hyperactivation of the PI3K/AKT/mTOR pathway. These molecules render standard endocrine therapies ineffective, highlighting the urgent need for alternative therapeutic approaches to overcome or delay resistance [5,6,7]. Additionally, acquired resistance to endocrine therapy is linked to mutations in ERα, such as Y537S and D538G somatic mutations, by stabilizing the activating function-2 binding conformation, often arising in metastatic cases post-aromatase inhibitor therapy. These mutations render the receptor constitutively active, promoting ligand-independent receptor activation, allowing cancer cells to proliferate despite estrogen depletion or ER antagonism [8,9].

Additionally, HER2 overexpression, typically associated with HER2-positive breast cancer, and is linked to endocrine therapy resistance by activating alternative growth-promoting pathways. Similarly, increased expression of membrane receptors such as FGFR (fibroblast growth factor receptor), IGF-1R (insulin-like growth factor receptor), and EGFR (epidermal growth factor receptor) contributes to endocrine resistance by bypassing ER signaling. These alterations drive ligand-independent activation of downstream signaling pathways, including the MAPK/ERK and CDK4/6 pathways, reinforcing the need for combination therapies to target multiple resistance mechanisms [10].

Although there are several advancements which have been made in breast cancer treatment to increase survival and quality of life [11], the issue of drug resistance has driven the exploration and use of other therapies along with endocrine therapies such as immunotherapeutic, targeted therapy, and chemotherapy agents. Immunotherapies aim to enhance the body’s immune response to cancer cells, as well as targeted radiotherapy and chemotherapeutic agents that specifically address cancer cells molecular and cellular characteristics. These approaches aim to circumvent resistance, improve patient outcomes, and offer more individualized treatment options [12,13].

Recent advances in immune-targeted therapy offer promising avenues to address endocrine resistance. Targeted therapies, including CDK4/6 inhibitors and PI3K/AKT/mTOR inhibitors, such as PI3K inhibitors (e.g., alpelisib) have been approved for treating endocrine-resistant ER+ breast cancer, particularly in PIK3CA-mutated cases. Additionally, mTOR inhibitors (e.g., everolimus) have been combined with endocrine therapy to overcome resistance by inhibiting downstream signaling [14]. Moreover, immune checkpoints, initially effective in triple-negative breast cancer, are now being explored in combination with endocrine therapy and targeted agents to enhance antitumor immune responses in HR+ breast cancer [15,16]. In addition, our group has also published a study in which we have demonstrated that combination therapy in drug resistance has better outcomes in breast cancer treatment [2].

The integration of targeted therapies with endocrine treatments has shown promise in delaying resistance and improving patient outcomes. Additionally, immune checkpoint inhibitors (ICIs), previously successful in triple-negative breast cancer, are now being explored in HR+ breast cancer to enhance antitumor immunity. Advances in combination therapies, leveraging immunotherapy, targeted agents, and chemotherapy, present a compelling avenue to manage drug-resistant cases. In this current study, we have discussed an overview of the molecular mechanism of endocrine resistance and the role of combination therapies in overcoming the drug resistance issue. Apart from this, we have also analyzed data from 35 randomized clinical trials to assess the impact of combinations of immunotherapy, targeted therapy, and chemotherapies in managing endocrine-resistant breast cancer, focusing on patients’ overall survival and progression-free survival. By synthesizing evidence from diverse therapeutic strategies, this study aims to provide a comprehensive understanding of the evolving treatment landscape, highlighting opportunities to improve outcomes in this challenging subset of breast cancer patients.

## 2. Molecular Mechanism of Endocrine Resistance

**Figure 1:**
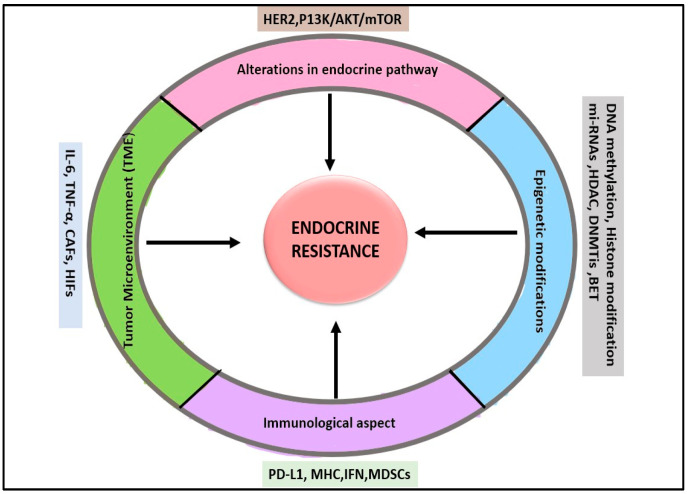
Schematic representation illustrating the factors contributing to endocrine resistance in breast cancer.

The molecular mechanisms of endocrine resistance in breast cancer are multifaceted. There are several reasons for resistance, such as alterations in estrogen receptor (ER) signaling, compensatory pathways, and the tumor microenvironment (Figure 1). An overview has been discussed below.

### 2.1. Alterations in the Endocrine Pathway

There are several factors for alteration pathways and acquired drug resistance, but the loss of estrogen receptor (ER) expression is one potential cause, with 10–20% of initially ER-positive patients transitioning to ER-negative upon relapse [17]. This loss of ER expression deprives cancer cells of their dependency on estrogen-driven proliferation, rendering therapies targeting ER signaling, such as selective estrogen receptor modulators (SERMs) or aromatase inhibitors, less effective. Another mechanism is the emergence of mutations in endocrine receptors [18]. Mutations such as ESR1 Y537S and D538G are among the most frequently observed alterations linked to the receptor’s constitutive activation. These mutations result in estrogen-independent signaling, allowing cancer cells to bypass the need for ligand binding. As a result, these mutations drive resistance to first-line endocrine therapies and pose significant challenges for subsequent treatment strategies [19].

**Figure 2:**
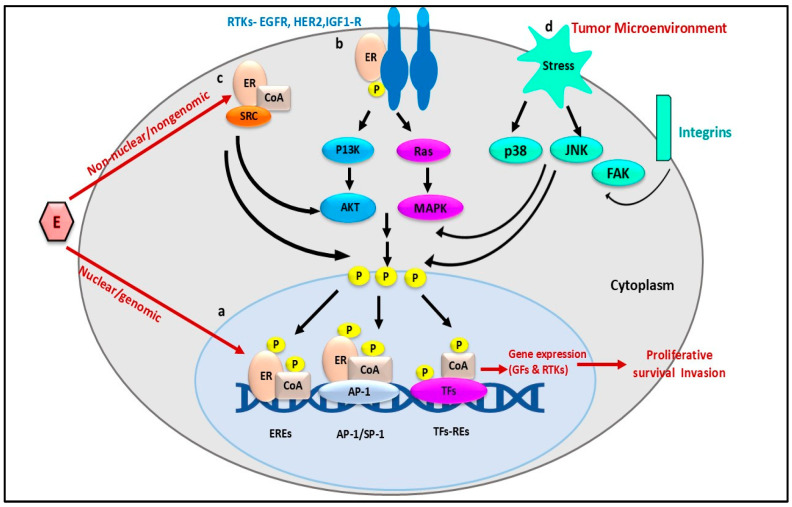
Illustrations represent the insights of an endocrine resistance mechanism. This diagram depicts the estrogen receptor (ER) signaling pathways in cancer, showing both nuclear/genomic and non-nuclear/nongenomic mechanisms. Estrogen (E) activates ERs, leading to gene expression via direct DNA binding or signaling cascades involving PI3K/AKT, Ras/MAPK, and stress-related pathways (p38, JNK, FAK). The tumor microenvironment influences these pathways, promoting proliferation, survival, and invasion. Phosphorylation plays a key role in activating transcription factors (TFs) and coactivators (CoA), enhancing gene expression of growth factors (GFs) and receptor tyrosine kinases (RTKs). This model is relevant for understanding estrogen receptor-positive cancers, particularly breast cancer, and identifying potential therapeutic targets. (Note: (a) denoting the nuclear/genomic mechanisms, (b) RTKs-EGFR, HER2, and IGF1-R mechanism, (c) non-nuclear/nongenomic mechanism, and (d) is showing tumor microenvironment for endocrine resistance).

Endocrine resistance in hormone receptor positive breast cancer presents a complex challenge influenced by various molecular changes, not just the loss of estrogen receptors (ER) or mutations. One significant factor contributing to this resistance is the increased activity of growth factor signaling pathways (HER2 and the PI3K/AKT/mTOR) which can drive tumor progression. These pathways promote cell survival, proliferation, and resistance to endocrine therapies. The dysregulation of the cell cycle machinery plays an important role in endocrine resistance. Alterations in proteins like cyclinD1(cdk), cyclin-dependent kinases (cdks), and tumor suppressors PRB1 and Tp53 can lead to uncontrolled cell division, diminishing the effectiveness of endocrine therapies. The interplay between ER signaling and other molecular networks adds further complexity, as shown in Figure 2. ER signaling is associated with pathways like p38, c-Jun N-terminal kinase (JNK), and focal adhesion kinase (FAK), which can compensate for ER inhibition and help sustain tumor growth. Specifically, the activation of the AKT/MAPK pathway through EGFR signaling can lead to the phosphorylation of ER or its co-regulators, allowing for ligand independent activation of ER target genes. To address endocrine resistance, combination therapies that target multiple pathways have emerged as a promising strategy. Dual inhibition approaches, such as combining endocrine therapy with PI3K/mTOR inhibitors have shown improved efficacy by blocking alternative survival mechanisms. Moreover, integrating CDK4/6 inhibitors with endocrine therapy has become a standard treatment option, significantly delaying disease progression in advanced hormone receptor-positive breast cancer [20].

### 2.2. Epigenetic Modifications

Epigenetic modifications have emerged as critical contributors to endocrine resistance in breast cancer, presenting significant challenges to the efficacy of therapies targeting estrogen receptor (ER) signaling. These modifications involve reversible alterations in gene expression without altering the underlying DNA sequence and play a pivotal role in reprogramming tumor cells to evade endocrine therapy [21].

DNA methylation is one of the most studied epigenetic changes associated with endocrine resistance. Hypermethylation of the promoter region of the ESR1 gene, which encodes the ER, leads to transcriptional silencing of ER expression. Loss of ER renders breast cancer cells unresponsive to therapies such as tamoxifen, fulvestrant, and aromatase inhibitors, which rely on the presence of functional ER [22]. Additionally, global hypomethylation, often observed in resistant tumors, can activate oncogenes and contribute to an aggressive phenotype. These methylation changes highlight the dynamic interplay between DNA methylation and resistance mechanisms [22].

Histone modifications, including acetylation, methylation, and phosphorylation, further contribute to endocrine resistance by altering chromatin structure and accessibility. Dysregulation of enzymes involved in histone modifications, such as histone deacetylases (HDACs), histone acetyltransferases (HATs), and histone methyltransferases (HMTs), disrupts the transcriptional regulation of ER and ER-associated genes [23]. For instance, increased HDAC activity in resistant cells leads to chromatin compaction and transcriptional repression of tumor suppressor genes. In contrast, aberrant HAT or HMT activity can promote the expression of genes driving resistance. The interplay between histone modifications and ER signaling emphasizes the complexity of epigenetic regulation in endocrine resistance. Another layer of epigenetic regulation involves chromatin remodeling complexes, which reorganize chromatin architecture at key regulatory loci. These complexes, such as the SWI/SNF family, alter nucleosome positioning and accessibility to transcription factors, including ER and its cofactors. Dysregulation of chromatin remodeling in resistant cells disrupts the ER-associated transcriptional network, further compromising the efficacy of endocrine therapies [24].

Non-coding RNAs, particularly microRNAs (miRNAs) and long non-coding RNAs (lncRNAs), play a crucial role in mediating epigenetic modifications associated with endocrine resistance. For example, miR-221 and miR-222, which are often upregulated in resistant breast cancers, target and downregulate tumor suppressor genes, such as p27 and p57, and modulate ER signaling pathways. Similarly, lncRNAs such as HOTAIR and MALAT1 regulate histone modification patterns and chromatin structure, promoting resistance by enhancing pro-survival and proliferative pathways. These non-coding RNAs act as epigenetic regulators, integrating with DNA methylation, histone modifications, and chromatin remodeling to drive resistance [25].

Targeting epigenetic modifications offers a promising approach to overcome endocrine resistance. Epigenetic modulators, together with HDAC inhibitors, DNA methyltransferase inhibitors (DNMTis), and bromodomain and extraterminal (BET) inhibitors, have shown potential in preclinical and clinical studies. These agents can reestablish ER expression, restart silenced tumor suppressor genes, and re-sensitize resistant tumors to endocrine therapies. For example, combining HDAC inhibitors with aromatase inhibitors or SERDs has proved to have synergistic effects in overcoming resistance. Similarly, DNMT inhibitors can reverse hypermethylation of the ESR1 promoter, restoring ER function and sensitivity to tamoxifen or fulvestrant [26].

Epigenetic modifications are central to the mechanisms underlying endocrine resistance in breast cancer. The interplay between DNA methylation, histone modifications, chromatin remodeling, and non-coding RNAs creates a complex regulatory network that drives resistance. Understanding these mechanisms provides insights into the biology of resistance and highlights novel therapeutic opportunities. Epigenetic-targeting agents, particularly in combination with endocrine therapies, hold great promise for improving outcomes in patients with resistant hormone receptor-positive breast cancer [27].

### 2.3. Tumor Microenvironment (TME)

The tumor microenvironment (TME) is one of the key factors for developing endocrine resistance in breast cancer through a supportive niche for tumor progression and therapy evasion. The TME comprises various cellular and non-cellular components, including stromal cells, immune cells, extracellular matrix, and secreted factors, collectively influencing tumor behavior. Hypoxia, a hallmark of the TME, drives resistance by stabilizing hypoxia-inducible factors (HIFs), which modulate gene expression to promote angiogenesis, metabolic reprogramming, and survival pathways. These HIF-mediated changes can bypass estrogen receptor (ER)-dependent signaling, reducing the efficacy of endocrine therapies [28]. However, inflammatory cytokines, such as interleukin-6 (IL-6) and tumor necrosis factor-alpha (TNF-α), secreted by immune and stromal cells, activate alternative signaling pathways, including the PI3K/AKT/mTOR and MAPK cascades, further diminishing ER dependence. [29]. Cancer-associated fibroblasts (CAFs), another critical component of the TME, secrete growth factors and extracellular matrix proteins that enhance cell proliferation and resistance mechanisms. Immune cells within the TME, such as tumor-associated macrophages (TAMs) and regulatory T cells (Tregs), suppress anti-tumor immunity and create an immunosuppressive environment that facilitates endocrine resistance [30]. Additionally, extracellular vesicles (EVs) released by TME components carry microRNAs, proteins, and metabolites that reprogram tumor and stromal cells, promoting resistance. Understanding the complex interactions within the TME and their impact on endocrine resistance highlights the need for novel therapeutic strategies [31]. Targeting the TME, either by inhibiting key components such as HIFs, cytokines, or CAFs, or by modulating immune checkpoints, combined with endocrine therapies, holds promise for overcoming resistance and improving treatment outcomes in hormone receptor-positive breast cancer [32].

## 3. Immunological Aspect of Endocrine Drug Resistance

There are two main factors in endocrine resistance. Intrinsic resistance dampens the effect of ICIs in certain situations, for instance, the mutation of PD-L1 or MHC molecules. The mutations abate the ability to present antigens that prevent the immune cells from identifying the cancer. Dysregulated interferon (IFN) signaling, weakened IFN signaling, deters activation of the immune and further undermines the immune assault against cancer [33]. The second factor is extrinsic resistance linked with the tumor microenvironment, which is the most critical factor in resistance, regulatory T cells (Tregs), and myeloid-derived suppressor cells (MDSCs); these immune cells suppress the anti-tumor immune response. Pro-inflammatory cytokines (e.g., IL-6, TGF-β) further suppress immunity and promote tumor progression. New technologies, such as TLA-based approaches paired with ICIs, aim to remodel the TME and enhance immune activation [34].

## 4. Role of Immune Immunotherapies in Combating Endocrine Resistance

Immunotherapy is developed via complex interactions between tumor cells and the immune system, creating an immunosuppressive TME that reduces the treatment’s efficacy [32]. Endocrine-resistant tumors often use the immune checkpoint pathways to avoid immune surveillance. The most common pathways are: (a) PD-1/PD-L1 axis: tumor cells make use of these pathways to switch off T-cells, thus, preventing immune attacks [35]. (b) The CTLA-4 pathway: this checkpoint dampens T-cell activation, further dampening the immune response. Combination therapies are now being explored to overcome these defenses. The combination of ICIs with hypoxia-targeting agents like TH-302 boosts T-cell activity by improving oxygenation and immune infiltration in low-oxygen tumor regions [36].

### Targeting Tumor-Associated Immune Cells and Cytokine

Emerging studies, such as single-cell RNA sequencing (scRNA-seq), describe the functions of immune cells, such as macrophages and neutrophils, in resistance. For instance, TAMs, tumor-associated macrophages, often support tumor growth and suppress immunity. The disruption of the CD47, a cell -surface protein expressed on cells that protects them from the immune system cells, reprograms TAMs to attack tumors and increases ICI efficacy. Modifying neutrophil behavior may further boost responses to ICIs and radiotherapy [35]. Endocrine-resistant cancers often secrete cytokines IL-6 and IL-8. These molecules trigger chronic inflammation and tumor progression. TGF-β, a cytokine, dampens immune responses and induces resistance. TGF-β blockers combined with endocrine treatments proved to reactivate the immune response and inhibit tumor advancement [36]. Apart from this, low tumor-infiltrating lymphocyte (TIL) counts in endocrine-resistant tumors are a significant challenge. Solutions include adoptive cell transfer, or ACT, whereby immune cells are transferred to the patient to directly enhance immune activity and direct cytokine therapies, administering pro-inflammatory cytokines to stimulate TIL recruitment and function [34]. These treatment strategies improve immune responses, making the tumor vulnerable to other ICIs and therapies.

## 5. Combination Strategies for Overcoming Resistance

Advanced therapies are being developed to address resistance, including CAR-T cells and engineered T-cells that specifically target tumor antigens [37]. ICIs targeting hypoxic regions in the TME show enhanced efficacy. Biomarker-driven approaches are essential to maximize success. These involve selecting therapies based on specific features of a patient’s tumor, ensuring personalized and effective treatments. Overcoming endocrine resistance in cancer requires innovative and multi-pronged approaches. Understanding and targeting intrinsic and extrinsic resistance mechanisms can optimize immunotherapy strategies, especially those combining ICIs with other modalities, to improve patient outcomes.

To check the impact of different therapies in combination, we carried out a meta-analysis demonstrating the significance of combination therapies to combat endocrine resistance; a detailed meta-analysis is mentioned below.

## 6. Materials and Methods

### 6.1. Search Strategy for Selection of Randomized Clinical Trials

A systematic review and meta-analysis were conducted using the Preferred Reporting Items for Systematic Reviews and Meta-analyses (PRISMA) criteria to ensure transparency, rigor, and consistency [2,38,39] (Supplementary Tables S4 and S5), and was not registered. The literature search was conducted via three databases: “Clinicaltrials.gov.in”, “PubMed”, and “Science Direct”, following PRISMA criteria. The keywords employed to locate the finalized research on “Clinicaltrials.gov.in” and PubMed were “Endocrine therapy”, “Targeted therapy”, and “Immunotherapy” in relation to “endocrine resistance breast cancer”.

The study was designed following the Patients, Intervention, Comparison, Outcome, and Study Design (PICOS) framework. As mentioned below:

Patients: The randomized clinical trials comprised breast cancer patients with endocrine resistance and a sensitive group.
Intervention(s): Studies were considered that used a combination of medicine therapy and targeted therapy or endocrine therapy with immunotherapy in endocrine resistance and sensitive groups.
Comparison(s): The study predominantly concentrated on overall survival (OS) as the primary endpoint. Secondary outcomes encompassed additional survival metrics, including progression-free survival (PFS).
Objective Measures: The main objective of the trial was overall survival (OS), whereas secondary goals encompassed progression-free survival (PFS).
Study design: The study strategy comprised exclusively randomized controlled trials (RCTs).

### 6.2. Data Acquisition

The two authors (AK and MA) conducted the screening of the studies according to the established inclusion and exclusion criteria and subsequently assessed their results. A conclusive choice was reached and juxtaposed with the perspective of a third author (SS). Only trials that provide statistical analysis for overall survival (OS) and progression-free survival (PFS) in patients receiving targeted therapy, immunotherapy chemotherapy, or in combination with additional agents (two or more) were considered.

### 6.3. Inclusion Criteria for Studies

Studies were considered to evaluate the outcomes of endocrine drug-resistant patients receiving treatment-targeted therapy or immunotherapy for breast cancer.

Furthermore, only studies that provided (a) statistical median values with 95% confidence intervals for overall survival (OS) and progression-free survival (PFS) were included.

### 6.4. Exclusion Criteria for the Studies

Studies were omitted based on established exclusion criteria, which are:

- Any replicated study.
- Research on conditions other than breast cancer.
- Results published were different from those on endocrine resistance in breast cancer.
- The studies lacked statistical median values and 95% confidence intervals.
- The studies lacked overall survival (OS) and progression-free survival (PFS) results.

### 6.5. Evaluation of Quality of Included Randomized Clinical Trials

The potential for bias in randomized controlled trials (RCTs) was assessed utilizing the Cochrane Collaboration’s Risk of Bias (RoB) tool within Review Manager software (version 5.3) (https://community.cochrane.org/help/tools-and-software/revman-5 accessed on 3 January 2025). The assessment included seven essential domains: random sequence generation (to identify selection bias), allocation concealment (to detect selection bias), blinding of participants and personnel (performance bias), blinding of outcome assessment (detection bias), incomplete outcome data (attrition bias), selective reporting (reporting bias), and additional biases (including funding sources). 

### 6.6. Statistical Analysis

The overall survival (OS) and progression-free survival (PFS) of patients receiving a combination of immunotherapies (with an additional agent or several immunotherapies) compared to those undergoing single immunotherapy were analyzed using statistical median values and 95% confidence intervals. The statistical data from our results was analyzed using the overall Risk Ratio (RR) and heterogeneity (I^2^ statistics) expressed as a percentage value. All statistical analyses were conducted using RevMan 5.3 software, with *p* < 0.05 deemed significant.

## 7. Results

### 7.1. Search Parameters and Study Selection

**Figure 3:**
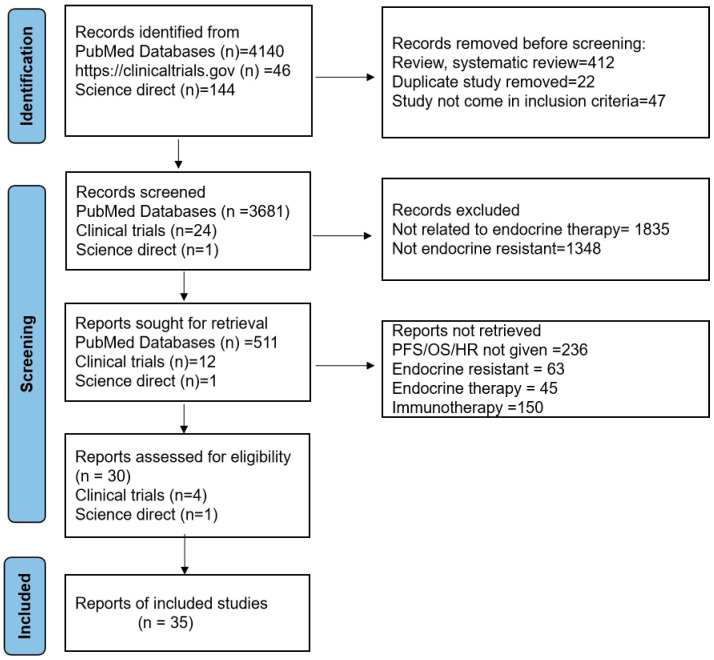
Prisma flowchart of the study selection process.

The preliminary search aimed to collect studies from different databases, and we found 4140 from PubMed, 46 from clinicaltrials.gov, and 144 from Science Direct (Figure 3). Using the established inclusion and exclusion criteria for eligibility and screening, 35 studies were chosen for the meta-analysis. Furthermore, studies were omitted if they lacked overall survival (OS) or progression-free survival (PFS) and 95% confidence intervals.

### 7.2. Impact of Other Therapies on Endocrine Resistance

**Figure 4:**
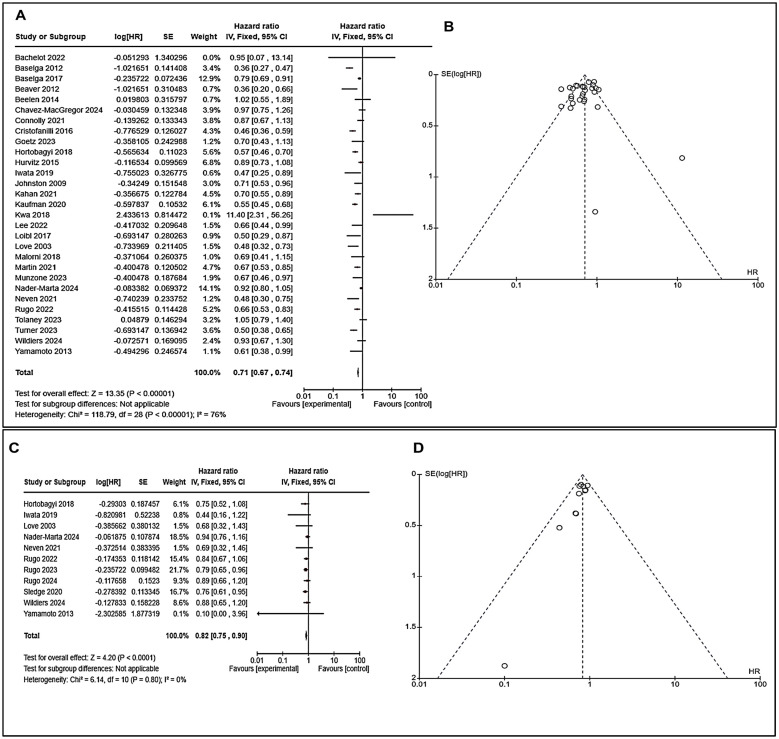
PFS and OS results for all selected studies were used for endocrine breast cancer with a hazard ratio (HR) and 95% confidence interval. (**A**) Forest plot for PFS: The pooled hazard ratio (HR) is 0.71 [0.67, 0.74], indicating a 29% reduced risk of disease progression with overall available therapies. However, heterogeneity is high (I^2^ = 76%), suggesting study variability. (**B**) Funnel plot for PFS: mostly symmetrical distribution, but some outliers suggest potential publication bias. (**C**) Forest plot for OS: The pooled HR is 0.82 [0.75, 0.90], indicating an 18% reduced risk of death. Low heterogeneity (I^2^ = 0%) suggests consistent results. Additionally, in one study, Yamamoto 2013 extends beyond the displayed range, suggesting high uncertainty. (**D**) Funnel plot for OS: symmetric, indicating minimal publication bias and strong reliability. (Details of these studies or groups are mentioned in Supplementary Tables S1–S5).

We pooled the analysis of different drugs with endocrine therapy or without endocrine therapy in endocrine resistance breast cancer. We have included all 35 studies (Supplementary Tables S1–S5) for this meta-analysis and observed a significant outcome in the form of progression-free survival with an overall effect Z = 13.35 (*p* < 0.00001) with HR 0.71 (CI: 0.67–0.74) and overall effect (Z) on overall survival was 4.20 (*p* < 0.0001) with HR 0.82 (CI: 0.75–0.90) (Figure 4). These results indicate that if we use other therapies in endocrine-resistant breast cancer patients, they may survive better.

### 7.3. Analysis of Targeted Therapies

**Figure 5:**
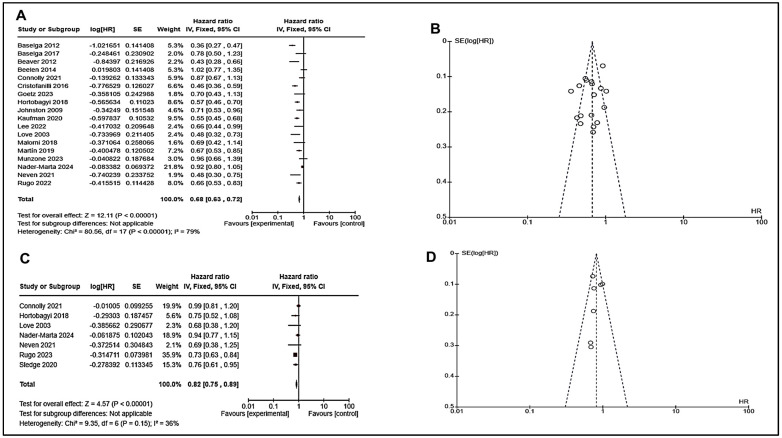
Analysis for targeted therapy for PFS and OS with hazard ratio (HR); 95% confidence interval (**A**) Forest plot for PFS indicated better survival with targeted therapy HR < 1 showing reduced risk of disease progression. (**B**) Funnel plot for PFS symmetric funnel plots indicate low publication bias. (**C**) Forest plot for OS. (**D**) Funnel plot for OS. The PFS analysis shows higher heterogeneity (I^2^ = 79%), while OS has lower variability (I^2^ = 36%), making OS findings more consistent. Overall, targeted therapy improves survival, as seen in HR < 1 for both PFS and OS, favoring the experimental group. (Details of these studies or groups are mentioned in Supplementary Table S1).

When we observed the significant results in pool studies for other therapies (targeted therapy, immunotherapy, and chemotherapies) used in endocrine resistance, we were curious to know which therapies have an impact on endocrine-resistant patients, and we included all studies of those on particular targeted therapy in endocrine resistance patients (Supplementary Table S1). We have observed that targeted therapy has significant results in terms of PFS and OS. The overall effect on progression-free survival was 12.11 (*p* < 0.0001) with HR 0.68 (CI: 063–0.72) and on overall survival Z-4.57 (*p* < 0.00001) with HR 0.82 (0.75–0.89) (Figure 5), which showed significant improvement in PFS and OS in endocrine resistance breast cancer patients.

### 7.4. Combined Effect of Immunotherapy and Endocrine Therapy

**Figure 6:**
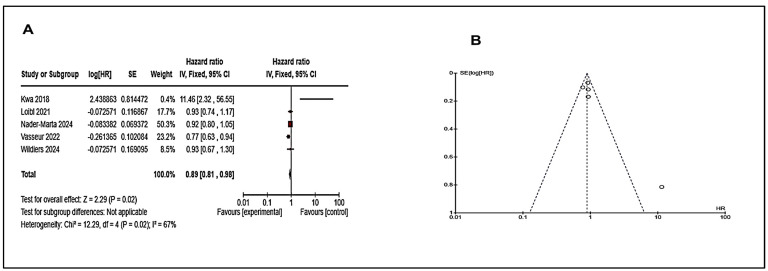
Analysis for Immunotherapy and Endocrine therapy for PFS with HR, OS with hazard ratio (HR); 95% confidence interval, (**A**) PFS forest plot shows a pooled hazard ratio (HR) of 0.89 [0.81, 0.98], indicating an 11% reduced risk in the experimental group and red box and dashes indicating the point estimate (or mean) of the effect size for a single study. However, heterogeneity is high (I^2^ = 67%), suggesting study variability. (**B**) Funnel plot for PFS shows slight asymmetry, indicating potential publication bias. Overall, targeted therapy provides moderate survival benefits.

We also analyzed the results for studies on the combined effect of immunotherapy and endocrine therapies (Supplementary Table S2) and noticed a PFS overall effect of 2.29 9 (*p* = 0.02) with HR 0.89 (0.81–0.98), which reflects little impact (Figure 6). We have not found studies having results for overall survival.

### 7.5. Analysis of Combined Therapies

**Figure 7:**
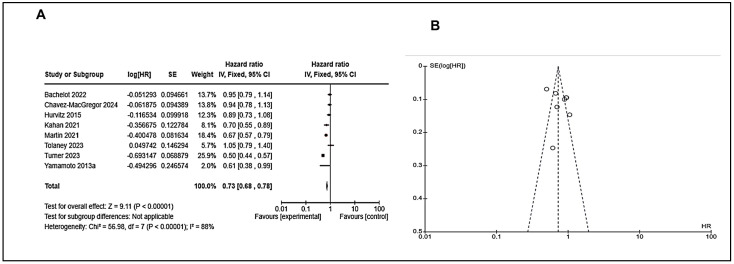
Analysis of combined therapies for PFS with hazard ratio (HR); 95% confidence interval, (**A**) PFS forest plot shows a pooled hazard ratio (HR) of 0.73 [0.68, 0.78], indicating a 27% reduced risk with combined therapies. However, high heterogeneity (I^2^ = 88%) suggests study variability. (**B**) Funnel plot for PFS is mostly symmetrical, indicating minimal publication bias. Overall, combined therapies significantly improve outcomes despite study differences. (Details of these studies or groups are mentioned in Supplementary Table S3).

To know other facts about the treatment of endocrine resistant breast cancer patients, we analyzed the studies which used combined therapies (immunotherapy/targeted therapy/chemotherapy in endocrine treatment (Supplementary Table S3). We observed that these combined therapies have a significant outcome in the form of progression-free survival with an overall effect of 9.11 (*p* < 0.00001) with HR 0.73 (0.68–0.78) (Figure 7). We did not find data for overall survival in this category.

In summary, the overall results found that using other therapies, such as targeted therapy, immunotherapy, and chemotherapies, may improve progression-free survival and overall survival. Targeted therapies showed significant improvements in progression-free survival (PFS) and overall survival (OS) in endocrine-resistant breast cancer patients. However, the combined effect of immunotherapy and endocrine therapy showed minimal impact on PFS and overall survival.

### 7.6. Risk of Bias Analysis

**Figure 8:**
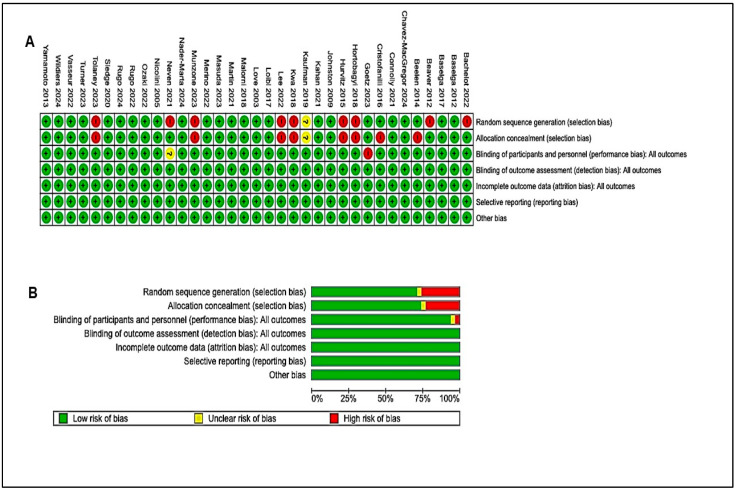
(**A**,**B**): Assessment of bias risk for the included studies utilizing the Cochrane Risk of Bias (RoB) tool within Review Manager software. (**A**) Graph depicting risk of bias. (**B**) Summary of risk of bias. Red indicates a high risk of bias, green signifies a low risk of bias, and yellow or blank denotes an unclear risk of bias. A plus sign denotes low risk of bias, a minus sign denotes high risk of bias, and a question mark denotes an unclear risk of bias. (Details of these studies or groups are mentioned in Supplementary Tables S1–S5).

Additionally, funnel plots for progression-free survival and overall survival (Figure 4B,D, Figure 5B,D, Figure 6B and Figure 7B) were examined to evaluate the publication biases of the study. Furthermore, we assessed the risk of bias utilizing the Cochrane Risk of Bias (RoB) tool within Review Manager software. We determined a minimal risk of bias for the eligible studies included (Figure 8A,B). The results of this study indicate that combination immunotherapies significantly enhance overall survival and progression-free survival outcomes compared to solo immunotherapy, with improved disease outcomes observed.

## 8. Discussion

In the current study, we reviewed one of the major issues in breast cancer, which is endocrine resistance, along with a meta-analysis of randomized clinical trials where we have observed significant results in combination therapies. Endocrine resistance is one of the significant challenges to treating receptor-positive breast cancer. Although several research studies are being done to overcome endocrine resistance for better treatment outcomes, which therapeutic strategy of therapeutics is effective in endocrine resistance is still not entirely known.

Some studies have suggested that a combination of other therapies may enhance the survival of endocrine resistance breast cancer. A systematic literature search was conducted by Schettini et al. to assess the efficacy of first-/second-line endocrine therapies in hormone receptor-positive/HER2-negative metastatic breast cancer. Thirty-five phase II/III randomized clinical trials (RCTs) were included, with results showing significant reductions in relapse or death risk. Combination strategies were more effective than single-agent endocrine therapy, with CDK4/6-inhibitors(i) plus endocrine therapy being the most effective regimen. These results strengthen international treatment guidelines and aid therapeutic decision-making [40]. Another study done by Brandao et al. demonstrated that when advanced breast cancer is treated with endocrine therapy (ET)-based treatments, it gives better progression-free survival (PFS) in endocrine-sensitive patients. In the endocrine-resistant setting, CDK4/6i + F500 was the most effective treatment, followed by capivasertib + F500 [41].

To check the impact of different combined therapies on endocrine resistance, we carried out a systematic review and meta-analysis on RCTs to investigate the effectiveness of different treatment strategies in endocrine resistance breast cancer patients. This systematic review and meta-analysis address critical advancements in immunotherapy and targeted therapies combined with endocrine therapy to overcome resistance in breast cancer treatment. In summary, our meta-analysis demonstrates that supplementary therapeutic strategies can markedly improve progression-free survival and, to a lesser degree, overall survival in cases of endocrine-resistant breast cancer. Targeted therapies and agents demonstrate significant efficacy as adjuncts to endocrine therapy. The role of immunotherapy is not yet fully understood and necessitates additional research to determine which patients may derive the most significant benefit.

Further analysis of the combined use of immunotherapy, targeted therapy, and chemotherapy with endocrine therapy demonstrated significant benefits in PFS, though OS data was limited. These results suggest that multi-modal treatment approaches may be a promising strategy to overcome the multifactorial nature of endocrine resistance. However, the heterogeneity in study designs and patient populations underscores the need for more robust clinical trials to validate these findings and establish standardized protocols. These findings align with recent studies, such as those by Schettini et al. and Brandao et al., which demonstrated the superior efficacy of combination strategies over monotherapy in endocrine-resistant HR+ breast cancer.

Our analysis contributes to the growing evidence that combining endocrine therapy with immunotherapy, targeted agents, and chemotherapy enhances therapeutic efficacy in patients with endocrine-resistant breast cancer. Future research should aim to clarify the molecular mechanisms underlying endocrine resistance and to enhance treatment sequencing and combinations to improve patient outcomes in this complex clinical context.

## 9. Limitations

This systematic review and meta-analysis relied solely on data from randomized controlled trials (RCTs), limiting the scope to studies that met stringent methodological criteria. Additionally, variances in study design, endpoints, and quality among the included studies may have impacted the consistency of the results. The data on the effectiveness of combining immunotherapy with targeted therapy across specific breast cancer subtypes and stages, particularly in HR+ and HER2-negative subgroups, were limited. This restriction hinders the generalizability of the findings across all patients with endocrine-resistant breast cancer. Variations in the combination and dosing of immunotherapies and targeted therapies among the studies may have introduced heterogeneity, affecting the comparability of outcomes. The study has limited outcome factors such as overall survival and progression-free survival only, and this lacks comprehensive outcomes such as Disease-Free Survival (DFS), Distant Metastasis-Free Survival (DMFS), Recurrence-Free Survival (RFS), Cause-Specific Survival (CSS), and Event-Free Survival (EFS) Although efforts were made to minimize publication bias, the possibility remains that studies with non-significant or negative results were underreported. This could lead to an overestimation.

Moreover, with demographic limitations, the findings may not fully apply to all patient populations, as most included studies were conducted in Western populations, with potential underrepresentation of data on Asian or other ethnic groups who may have different responses to therapy. Despite these limitations, the analysis provides a valuable assessment of immunotherapy and targeted therapy in endocrine-resistant breast cancer. It highlights the need for larger, well-designed studies to validate these findings across diverse patient populations and subtypes.

## 10. Conclusions

Endocrine resistance continues to be a major and significant obstacle in the management of hormone receptor breast cancer, diminishing the effectiveness of conventional endocrine therapies and resulting in poor clinical outcomes for many patients due to its resistance. Our current metanalysis provides evidence that integration of other therapies such as immunotherapy, targeted therapy, and chemotherapy, with endocrine therapy may offer promising results in aspects of endocrine resistance. We carried out a pooled analysis of all types of combination therapies for 35 randomized control trials, which demonstrated that these combination strategies significantly improved progression-free survival and overall survival in patients with endocrine resistance. When combined with endocrine therapy, immunotherapy exhibited limited but encouraging outcomes, highlighting the potential need for patient stratification to identify those who might benefit most. The combined use of multiple therapeutic modalities further underscored the value of a multifaceted approach, although long-term data on survival outcomes remain sparse.

### Future Prospects

The prospects for overcoming endocrine resistance in breast cancer are promising, with ongoing research focused on understanding the molecular mechanisms underlying resistance and developing novel therapeutic strategies. Several emerging areas are showing great potential to address endocrine resistance in hormone receptor-positive (HR+) breast cancer, improving both survival outcomes and quality of life. The future of overcoming endocrine resistance in breast cancer lies in the development of personalized, combination-based treatment strategies that target both the estrogen receptor and alternative resistance pathways. Emerging therapies, including oral SERDs, CDK4/6 inhibitors, PI3K and mTOR inhibitors, immune checkpoint inhibitors, and employing multi-pronged approaches—such as integrating novel epigenetic modulators—offer new hope for patients who develop resistance to current endocrine therapies. Additionally, the use of precision medicine, liquid biopsies, and AI-driven approaches will enhance the ability to tailor treatment plans and monitor disease progression in real time. However, to fully harness the benefits of AI in clinical practice, it is imperative to establish standardized guidelines and promote trustworthy AI practices. These measures will ensure fairness, traceability, and robustness, ultimately facilitating the integration of AI technologies into routine breast cancer care [42,43].

## Data Availability

The data from randomized control trials used and/or analyzed during the current study are available in Supplementary Tables, and any other row data will be provided by the corresponding author upon reasonable request.

## Supplementary Materials

The following supporting information can be downloaded at: https://www.mdpi.com/article/10.3390/cancers17101653/s1, Table S1: Studies selected for meta-analysis for target therapies. Table S2: Endocrine therapy + Immunotherapy studies included in the analysis. Table S3: Combination therapies with Immunotherapy/Chemotherapy + Targeted therapy/Endocrine therapy included in the analysis. Table S4: PRISMA 2020 Main Checklist. Table S5: PRISMA Abstract Checklist. Reference [44] was cited in Supplementary Materials.

## References

[1] Sisodiya S., Kasherwal V., Khan A., Roy B., Goel A., Kumar S., Arif N., Tanwar P., Hussain S. (2023). Liquid Biopsies: Emerging role and clinical applications in solid tumours. Transl. Oncol..

[2] Sisodiya S., Kasherwal V., Rani J., Mishra N., Kumar S., Khan A., Aftab M., Shagufta, Singh P., Gupta E. (2024). Impact of combinatorial immunotherapies in breast cancer: A systematic review and meta-analysis. Front. Immunol..

[3] Bray F., Laversanne M., Sung H., Ferlay J., Siegel R.L., Soerjomataram I., Jemal A. (2024). Global cancer statistics 2022: GLOBOCAN estimates of incidence and mortality worldwide for 36 cancers in 185 countries. CA Cancer J. Clin..

[4] Lloyd M.R., Wander S.A., Hamilton E., Razavi P., Bardia A. (2022). Next-generation selective estrogen receptor degraders and other novel endocrine therapies for management of metastatic hormone receptor-positive breast cancer: Current and emerging role. Ther. Adv. Med. Oncol..

[5] Osborne C.K., Schiff R. (2011). Mechanisms of endocrine resistance in breast cancer. Annu. Rev. Med..

[6] Manohar P.M., Davidson N.E. (2021). Updates in endocrine therapy for metastatic breast cancer. Cancer Biol. Med..

[7] Rascio F., Spadaccino F., Rocchetti M.T., Castellano G., Stallone G., Netti G.S., Ranieri E. (2021). The Pathogenic Role of PI3K/AKT Pathway in Cancer Onset and Drug Resistance: An Updated Review. Cancers.

[8] Ladd B., Mazzola A.M., Bihani T., Lai Z., Bradford J., Collins M., Barry E., Goeppert A.U., Weir H.M., Hearne K. (2016). Effective combination therapies in preclinical endocrine resistant breast cancer models harboring ER mutations. Oncotarget.

[9] Rani A., Stebbing J., Giamas G., Murphy J. (2019). Endocrine Resistance in Hormone Receptor Positive Breast Cancer-From Mechanism to Therapy. Front. Endocrinol..

[10] Mazumder A., Shiao S., Haricharan S. (2021). HER2 Activation and Endocrine Treatment Resistance in HER2-negative Breast Cancer. Endocrinology.

[11] Yu J., Mu Q., Fung M., Xu X., Zhu L., Ho R.J.Y. (2022). Challenges and opportunities in metastatic breast cancer treatments: Nano-drug combinations delivered preferentially to metastatic cells may enhance therapeutic response. Pharmacol. Ther..

[12] Mukherjee A.G., Wanjari U.R., Namachivayam A., Murali R., Prabakaran D.S., Ganesan R., Renu K., Dey A., Vellingiri B., Ramanathan G. (2022). Role of Immune Cells and Receptors in Cancer Treatment: An Immunotherapeutic Approach. Vaccines.

[13] Bondhopadhyay B., Sisodiya S., Chikara A., Khan A., Tanwar P., Afroze D., Singh N., Agrawal U., Mehrotra R., Hussain S. (2020). Cancer immunotherapy: A promising dawn in cancer research. Am. J. Blood Res..

[14] Vernieri C., Corti F., Nichetti F., Ligorio F., Manglaviti S., Zattarin E., Rea C.G., Capri G., Bianchi G.V., de Braud F. (2020). Everolimus versus alpelisib in advanced hormone receptor-positive HER2-negative breast cancer: Targeting different nodes of the PI3K/AKT/mTORC1 pathway with different clinical implications. Breast Cancer Res..

[15] Ye F., Dewanjee S., Li Y., Jha N.K., Chen Z.S., Kumar A., Vishakha, Behl T., Jha S.K., Tang H. (2023). Advancements in clinical aspects of targeted therapy and immunotherapy in breast cancer. Mol. Cancer.

[16] Abdou Y., Goudarzi A., Yu J.X., Upadhaya S., Vincent B., Carey L.A. (2022). Immunotherapy in triple negative breast cancer: Beyond checkpoint inhibitors. NPJ Breast Cancer.

[17] Ors A., Chitsazan A.D., Doe A.R., Mulqueen R.M., Ak C., Wen Y., Haverlack S., Handu M., Naldiga S., Saldivar J.C. (2022). Estrogen regulates divergent transcriptional and epigenetic cell states in breast cancer. Nucleic Acids Res..

[18] Bedard P.L., Freedman O.C., Howell A., Clemons M. (2008). Overcoming endocrine resistance in breast cancer: Are signal transduction inhibitors the answer?. Breast Cancer Res. Treat..

[19] Fanning S.W., Mayne C.G., Dharmarajan V., Carlson K.E., Martin T.A., Novick S.J., Toy W., Green B., Panchamukhi S., Katzenellenbogen B.S. (2016). Estrogen receptor alpha somatic mutations Y537S and D538G confer breast cancer endocrine resistance by stabilizing the activating function-2 binding conformation. Elife.

[20] Miller T.W., Balko J.M., Arteaga C.L. (2011). Phosphatidylinositol 3-kinase and antiestrogen resistance in breast cancer. J. Clin. Oncol..

[21] Hussain S., Tulsyan S., Dar S.A., Sisodiya S., Abiha U., Kumar R., Mishra B.N., Haque S. (2022). Role of epigenetics in carcinogenesis: Recent advancements in anticancer therapy. Semin. Cancer Biol..

[22] Martinez-Galan J., Torres-Torres B., Nunez M.I., Lopez-Penalver J., Del Moral R., Ruiz De Almodovar J.M., Menjon S., Concha A., Chamorro C., Rios S. (2014). ESR1 gene promoter region methylation in free circulating DNA and its correlation with estrogen receptor protein expression in tumor tissue in breast cancer patients. BMC Cancer.

[23] Hervouet E., Cartron P.F., Jouvenot M., Delage-Mourroux R. (2013). Epigenetic regulation of estrogen signaling in breast cancer. Epigenetics.

[24] Vietri M.T., D’Elia G., Benincasa G., Ferraro G., Caliendo G., Nicoletti G.F., Napoli C. (2021). DNA methylation and breast cancer: A way forward (Review). Int. J. Oncol..

[25] Kumar S., Gonzalez E.A., Rameshwar P., Etchegaray J.P. (2020). Non-Coding RNAs as Mediators of Epigenetic Changes in Malignancies. Cancers.

[26] Yin J., Gu T., Chaudhry N., Davidson N.E., Huang Y. (2023). Epigenetic modulation of antitumor immunity and immunotherapy response in breast cancer: Biological mechanisms and clinical implications. Front. Immunol..

[27] Garcia-Martinez L., Zhang Y., Nakata Y., Chan H.L., Morey L. (2021). Epigenetic mechanisms in breast cancer therapy and resistance. Nat. Commun..

[28] Khalaf K., Hana D., Chou J.T., Singh C., Mackiewicz A., Kaczmarek M. (2021). Aspects of the Tumor Microenvironment Involved in Immune Resistance and Drug Resistance. Front. Immunol..

[29] Roy T., Boateng S.T., Uddin M.B., Banang-Mbeumi S., Yadav R.K., Bock C.R., Folahan J.T., Siwe-Noundou X., Walker A.L., King J.A. (2023). The PI3K-Akt-mTOR and Associated Signaling Pathways as Molecular Drivers of Immune-Mediated Inflammatory Skin Diseases: Update on Therapeutic Strategy Using Natural and Synthetic Compounds. Cells.

[30] Wu F., Yang J., Liu J., Wang Y., Mu J., Zeng Q., Deng S., Zhou H. (2021). Signaling pathways in cancer-associated fibroblasts and targeted therapy for cancer. Signal Transduct. Target. Ther..

[31] Lopez K., Lai S.W.T., Lopez Gonzalez E.J., Davila R.G., Shuck S.C. (2023). Extracellular vesicles: A dive into their role in the tumor microenvironment and cancer progression. Front. Cell Dev. Biol..

[32] Xu J., Gan C., Yu S., Yao S., Li W., Cheng H. (2024). Analysis of Immune Resistance Mechanisms in TNBC: Dual Effects Inside and Outside the Tumor. Clin. Breast Cancer.

[33] Nagasaki J., Inozume T., Sax N., Ariyasu R., Ishikawa M., Yamashita K., Kawazu M., Ueno T., Irie T., Tanji E. (2022). PD-1 blockade therapy promotes infiltration of tumor-attacking exhausted T cell clonotypes. Cell Rep..

[34] Tang L., Wang D., Hu T., Lin X., Wu S. (2024). Current applications of tumor local ablation (TLA) combined with immune checkpoint inhibitors in breast cancer treatment. Cancer Drug Resist..

[35] Lau A.P.Y., Khavkine Binstock S.S., Thu K.L. (2023). CD47: The Next Frontier in Immune Checkpoint Blockade for Non-Small Cell Lung Cancer. Cancers.

[36] Peng D., Fu M., Wang M., Wei Y., Wei X. (2022). Targeting TGF-beta signal transduction for fibrosis and cancer therapy. Mol. Cancer.

[37] Zheng Y., Li S., Tang H., Meng X., Zheng Q. (2023). Molecular mechanisms of immunotherapy resistance in triple-negative breast cancer. Front. Immunol..

[38] Moher D., Liberati A., Tetzlaff J., Altman D.G., Group P. (2010). Preferred reporting items for systematic reviews and meta-analyses: The PRISMA statement. Int. J. Surg..

[39] Janani M., Poorkhani A., Amiriani T., Donyadideh G., Ahmadi F., Jorjanisorkhankalateh Y., Beheshti-Nia F., Kalaei Z., Roudbaraki M., Soltani M. (2024). Association of future cancer metastases with fibroblast activation protein-α: A systematic review and meta-analysis. Front. Oncol..

[40] Schettini F., Giuliano M., Giudici F., Conte B., De Placido P., Venturini S., Rognoni C., Di Leo A., Locci M., Jerusalem G. (2021). Endocrine-Based Treatments in Clinically-Relevant Subgroups of Hormone Receptor-Positive/HER2-Negative Metastatic Breast Cancer: Systematic Review and Meta-Analysis. Cancers.

[41] Brandao M., Maurer C., Ziegelmann P.K., Ponde N.F., Ferreira A., Martel S., Piccart M., de Azambuja E., Debiasi M., Lambertini M. (2020). Endocrine therapy-based treatments in hormone receptor-positive/HER2-negative advanced breast cancer: Systematic review and network meta-analysis. ESMO Open.

[42] Diaz O., Rodriguez-Ruiz A., Sechopoulos I. (2024). Artificial Intelligence for breast cancer detection: Technology, challenges, and prospects. Eur. J. Radiol..

[43] Sarno F., Benincasa G., List M., Barabasi A.L., Baumbach J., Ciardiello F., Filetti S., Glass K., Loscalzo J., Marchese C. (2021). Clinical epigenetics settings for cancer and cardiovascular diseases: Real-life applications of network medicine at the bedside. Clin. Epigenetics.

[44] Page M.J., McKenzie J.E., Bossuyt P.M., Boutron I., Hoffmann T.C., Mulrow C.D., Shamseer L., Tetzlaff J.M., Akl E.A., Brennan S.E. (2021). The PRISMA 2020 statement: An updated guideline for reporting systematic reviews. BMJ.

