# Supplementary material for: Mechanisms and Therapeutic Strategies for Endocrine Resistance in Breast Cancer: A Comprehensive Review and Meta-Analysis
Source: Cancers (Basel). 2025 May 14;17(10):1653. doi: 10.3390/cancers17101653 (PMC12109706; doi:10.3390/cancers17101653)
Supplement: Supplementary file 1 [file cancers-17-01653-s001.zip › cancers-3482520-supplementary.pdf]

Mechanisms and Therapeutic Strategies for Endocrine Resistance in Breast Cancer: A Comprehensive Review and Meta-Analysis

Asiya Khan [1,2], Sandeep Sisodiya [3,4], Mehreen Aftab [3], Pranay Tanwar [5], Showket Hussain [3,*] and Vivek Gupta [2,*]

[1] Multidisciplinary Research Unit, Government Institute of Medical Sciences, Greater Noida 201310, India; ak.asiyakhan1@gmail.com
[2] Department of Pathology, Government Institute of Medical Sciences, Greater Noida 201310, India
[3] Cellular and Molecular Diagnostics (Molecular Biology Group), ICMR—National Institute of Cancer Prevention and Research, Ministry of Health and Family Welfare, Government of India, Noida 201301, India; sandeepsisodiya99@gmail.com (S.S.); mehreen_aftab2004@yahoo.com (M.A.)
[4] Symbiosis School of Biological Sciences (SSBS), Symbiosis International (Deemed University) (SIU), Pune 412115, India
[5] Lab Oncology Unit, Dr. B.R.A. Institute Rotary Cancer Hospital, All India Institute of Medical Sciences, New Delhi 110029, India; pranaytanwar@gmail.com
* Correspondence: showket.hussain@gov.in (S.H.); dr_vivek_gupta@yahoo.com (V.G.)

Supplementary Table S1: Studies selected for meta-analysis for target therapies.

S. No.	PMID	First Author	Publication Year	NCT number	Trial Phase	No. of patients	Age (median, range)	Targeted therapy	Hormone receptor status, n (%)	PFS/EFS Median; 95% CI interval	OS/ORR Median; 95% CI interval	Outcome
1.	36027558	Rugo S Hope	2022	NCT03901339	phase III	543	56	sacituzumab govitecan	HR+<br>HER2-	HR, 0.66 [95% CI, 0.53 to 0.83; p = .0003]	HR, 0.84; p = .14	SG demonstrated a significant PFS advantage over chemotherapy, with a manageable safety profile in heavily pretreated, endocrine-resistant HR+/HER

												2-advanced breast cancer patients.
2.	26947331	Cristofanilli M	2016	NCT01942135	Phase III	259	≥18	fulvestrant plus palbociclib	HR+ HER2-	9·5 months (95% CI 9·2-11·0) in the fulvestrant plus palbociclib group and 4·6 months (3·5-5·6) in the fulvestrant plus placebo		Fulvestrant and palbociclib improve progression-free survival compared to fulvestrant plus placebo. This combination is a potential treatment for recurrent

										group (HR 0·46, 95% CI 0·36-0·59, p<0·0001).		hormone-receptor-positive, HER2-negative metastatic breast cancer.
3.	29718092	Hortobagyi GN	2018	NCT01958021	Phase III	243	≥18	Ribociclib plus Letrozole	HR+, HER-	Median PFS was 25.3 months [95% confidence interval (CI) 23.0-30.3] for ribociclib plus letrozole and 16.0 months (95% CI	HR 0.746; 95% CI 0.517-1.078	The improved effectiveness and tolerable side effects of first-line ribociclib plus letrozole continue with longer follow-up compared to letrozole alone.

										13.4-18.2) for placebo plus letrozole (HR 0.568; 95% CI 0.457-0.704; log-rank p = 9.63 × 10-8)..		
4.	31563959	Sledge GW Jr	2019	NCT02107703	Phase III	669	59 [32 - 91]	Abemaciclib Plus Fulvestran	HR+, HER -	median, 23.1 months vs 20.6 month	a median OS of 46.7 months for abemaciclib plus fulvestrant and 37.3 months	Abemaciclib plus fulvestrant improved median OS by 9.4 months in HR-positive, ERBB2-negative advanced breast

											for placebo plus fulvestrant [HR], 0.757; 95% CI, 0.606-0.945; p= .01).	cancer patients who progressed after prior endocrine therapy. It also delayed the need for subsequent chemotherapy.
5.	22149876	Baselga J	2012	NCT00863655	phase III	34	62	everolimus and exemestane	HR+<br>HER2-	10.6 months and 4.1 months according to central assessment (HR, 0.36; 95% CI, 0.27 to 0.47; P<0.001).		Everolimus with an aromatase inhibitor improved progression-free survival in HR positive advanced breast cancer patients previously treated

												with nonsteroidal aromatase inhibitors.
6.	**37440239**	Munzone E,	2023	NCT02954055	Phase II	133	61(30-80)	oral vinorelbine plus cyclophosphamide plus capecitabine (VEX) regimen vs weekly IV paclitaxel	HR+ HER2-	The median PFS was 11.1 (95% CI, 8.3-13.8) months vs 6.9 (95% CI, 5.4-10.1) months favoring VEX (HR, 0.67; 95% CI, 0.46-		The trial indicated that oral VEX improved TTF and PFS over intravenous paclitaxel, but OS was unchanged. VEX may offer better disease control for ER+/ERBB2-metastatic breast cancer.

										0.96, p = .03).		
7.	38072514	Goetz MP	2023	NCT03781063	Phase II	103		Lasofoxifene versus fulvestrant	HR+ HER2-	24.2 weeks (~5.6 months) versus 16.2 weeks (~3.7 months; p = 0.138); HR 0.699 (95% confidence interval 0.434-1.125)		Lasofoxifene showed promising antitumor activity and was well tolerated in patients with ESR1-mutated metastatic breast cancer after prior treatments. It reduced ESR1 mutations more than fulvestrant and warrants further study as a

												targeted therapy.
8.	38123789	Rugo HS	2024	NCT02258464	Phase II	382		Radium-223 endocrine therapy versus placebo plus endocrine therapy	HR+ HER2-	0.809 (0.610-1.072), p = 0.1389	0.889 (0.660-1.199), p = 0.4410	
9.	35124320	Lee S	2022		Phase II	178	44 (31–58)	Palbociclib plus endocrine therapy	HR+ HER2-	median PFS of 20.1 months in the palbociclib plus ET versus 14.4 months in the capecitabine (HR 0.659 [95% CI		Palbociclib plus endocrine therapy has better outcomes for luminal types, while BRCA2 mutations lead to worse prognosis. More research is needed on biomarkers

										0.437–0.994], log-rank p = 0.0235)		for efficacy and resistance.
10.	28652278	Loibl S	2017	NCT01942135	Phase III	72+36	≥18	Palbociclib plus Fulvestrant	HR+ HER2-	palbociclib (*n* = 72) versus placebo arm (*n* = 36) was 9.5 versus 5.6 months, (HR, 0.50, 95% confidence interval: 0.29-0.87)		Palbociclib with fulvestrant and goserelin is effective for premenopausal women with resistant HR+/HER2- breast cancer. Trials should include both premenopausal and postmenopausal

												women to improve access to treatments.
11	29893 790	Malorn i L	2018	NCT02549430	Pha se II	115	≥18	Palbociclib plus endocrine therapy	HR+ HER2-	6.5 months (95% CI: 5.4-8.5) (HR) 0.69; 95% CI: 0.4-1.1,		Palbociclib with fulvestrant and goserelin is effective for premenopa usal women with resistant HR+/HER 2- breast cancer. Trials should include both premenopa usal and postmenop ausal women to improve

												access to treatments.
12.	**24447434**	Beelen K	2014			739	65		HR+ HER2-	tamoxifen (HR 0.24, P < 0.0001), while patients whose tumor did express p-p70S6K did not (HR = 1.02, P =0.95), P for interaction 0.004.		Patients with p-p70S6K tumors have a good prognosis but do not benefit from tamoxifen; further study on PI3K/Akt/mTOR inhibitors is needed.
13.	34357781	Connolly RM	2021	NCT02115282	Phase III		63 years (range 29-91	Endocrine Therapy Plus Entinostat or Placebo	HR+ HER2-	Median PFS was 3.3 months (EE) versus	Median OS was 23.4 months (EE) versus	The pairing of exemestane and entinostat did not

										3.1 months (EP; HR = 0.87; 95% CI, 0.67 to 1.13; *P* = .30).	21.7 months (EP; HR = 0.99; 95% CI, 0.82 to 1.21; *P* = .94).	enhance survival rates in advanced HR-positive, HER2-negative breast cancer that is resistant to aromatase inhibitors.
14.	32043763	Kaufman PA	2019	NCT02107703		855		Abemaciclib plus Fulvestrant	HR+ HER2-	HR, 0.55; 95% confidence interval [CI], 0.45–0.68; *p* < .001		HRQoL was maintained with abemaciclib plus fulvestrant, which showed superior PFS and manageable safety. These results

												support its use in endocrine-resistant HR+, HER2-negative ABC patients.
15.	31276981	Martín M	2019	NCT00545077 and NCT00601900	phase III	749	≥18	bevacizumab to endocrine therapy	HR+ HER2-	Median PFS was 14.3 months for ET versus 19 months for ET + Bev		[HR] 0.77; 95% confidence interval [CI] 0.66-0.91; p < 0.01).
16.	34425869	Neven P	2023	NCT02107703	Phase III	669	32-66	Abemaciclib plus fulvestrant	HR+ HER2-	28.6 months for abemaciclib plus fulvestrant versus	HR 0.689; 95% CI 0.379-1.252, median, not reached	The MONARCH 2 trial showed that abemaciclib plus fulvestrant (with

										10.26 months for placebo plus fulvestrant (HR 0.477; 95% CI 0.302-0.755).	vs 47.3 months	ovarian suppression) is effective for ET-resistant premenopausal patients with HR+, HER2- advanced breast cancer.
17.	19786658	Johnston S	2009	NCT00073528	863		59-63	Lapatinib combined with letrozole	HR+<br>HER2-	8.2 v 3.0 months, [HR] = 0.71; 95% CI, 0.53 to 0.96; P = .019)		This study found that the combination of letrozole and lapatinib significantly improves progression-free survival and clinical

												benefit in patients with HER2-positive, hormone receptor-positive metastatic breast cancer.
18.	12560434	Love RR	2003		282			Tamoxifen	HR+ HER2-	HR=0.48 (95% CI, 0.31 to 0.71).	HR=0.68 (95% CI, 0.32 to 1.42)	HER-2/neu overexpression may positively influence response to adjuvant oophorectomy and tamoxifen in estrogen receptor-positive tumors.
19.	22764762	Beaver JA	2012			PhaseIII		everolimus plus exemestane	HR+ HER2-	6.9 months for everoli		This research indicates that

										mus plus exemestane versus 2.8 months for placebo plus exemestane (HR: 0.43; p < 0.001)		combining everolimus with exemestane may be a promising treatment alternative for this group of patients.
20.	28576675	Baselga J,	2017	NCT01610284		546	≥18	Buparlisib plus fulvestrant	HR+ HER2-	6·9 months (95% CI 6·8-7·8) in the buparlisib group versus 5·0 months (4·0-		This study shows that PI3K inhibition with endocrine therapy is effective for postmenopausal women with

										5·2) in the placebo group [HR] 0·78 [95% CI 0·67-0·89]; one-sided p=0·00 021)		endocrine-resistant advanced breast cancer. More selective PI3K inhibitors may enhance safety and benefits.
21.	39375 745	Nader-Marta G	2024	NCT02513394	Pha se III	5753	53.1	Palbociclib plus endocrine therapy	ER + /H ER2-	82.1% HR 0.92 (0.80 – 1.05)	92.5% HR:0.9 4 (0.76 – 1.16)	no differences were observed in clinical parameters , prognosis, or differential benefit from palbociclib between HER2-0 and HER2-

												low tumors.

Supplementary Table S2. Endocrine therapy + Immunotherapy studies included in the analysis.

S. No.	PMID	First Author	Publication Year	NCT number	Trial Phase	No. of patients	Age (median, range)	Anti PD-1/ PD-L1 agent	Hormone receptor status, n (%)	PFS/EFS	OS/ORR Median; 95% CI interval	Outcome
1.	35728379	Ozaki Y	2022	WJOG9917B	Phase II	57	49 (31–76)	nivolumab plus bevacizumab, paclitaxel	HR+/HER2-	The median PFS were 14.0 (95% CI 11.0–16.3) and Grade 3/4 adverse drug reactions occurred in 33 of 57 patients (58%).	OS 32.5 (95% CI 26.0–not evaluable) months, respectively (median follow-up: 29.5 months).	Nivolumab, bevacizumab, and paclitaxel as first-line therapy showed promising effectiveness and manageable side effects in patients with HER2-negative metastatic breast cancer.

										Immune-related adverse events occurred in 43 of 57 patients (75%), with grade 3/4 events in eight patients (14%)	ORR 70%, [95% CI 55.9–81.2%], ORR74% in patients with HR-positive breast cancer versus 59% in patients with triple-negative breast cancer.	
2.	37939105	Wildiers H	2024	NCT02614833	Phase IIb	227	60 (24-87)	Paclitaxel<br>Eftilagimod alpha	HR+/HER2-	7.3 months [95% confiden	Median OS was 20.4	AIPAC did not achieve its PFS goal but confirmed efti's

										ce interval (CI), 6.6–7.5] in the efti arm and 7.3 months (95% CI, 5.5–7.5) in the placebo arm (HR, 0.93; 95% CI, 0.67–1.30; *P* = 0.341	months (95% CI, 14.3–25.1) in the efti arm and 17.5 months (95% CI, 12.9–21.8) in the placebo arm (HR, 0.88; 95% CI, 0.64–1.19; *P* = 0.197	pharmacodynamics and safety. OS showed a 2.9-month difference overall but improved in specific biomarker subgroups, suggesting further study in ET-resistant HER2-negative MBC.
3.	**36463104**	Merino et al	• 20	NCT03025880	Phase II	14	31-77	Pembrolizumab plus	ER+ HER2-	PFS was 3.1 months (95%	OS was 7.9 mo	The RP2D was pembrolizumab 200 mg and

			2 2					gemcitabine		CI 2.0–4.3)	nths (95% CI 6.5–10.3). ORR was 15% (95% CI, 5–32)<br>median	gemcitabine 1,250 mg/m2. ORR wasn't met, but 22% stayed on treatment for over 6 months. Effective biomarker selection is crucial for ABC patients, but TILs, PD-L1, and MDSCs did not identify those likely to benefit from the combination.
4.	29124456	Kwa M	• 2 0 1 8	NCT01963481	Phase II	23		exemestane with cyclophosphamide	HR2+/HER2-	Three-month PFS rate was 50.1% (95% CI 33.0-76.0%); median PFS was	OR were seen in 6/23 patients (26.1%; 95% CI	Treg depletion was not seen with low-dose cyclophosphamide, but baseline naïve Tregs were linked to 3-month PFS. Exemestane/cy

										4.23 months (95% CI 2.8-11.7). HR; 11.46 (95% CI 2.32-56.5)]	10.2-48.4%) and were durable (median 11.6 months).	clophosphamide showed good safety and clinical activity in heavily pretreated patients.
5.	https://doi.org/10.1016/j.breast.2022.07.014	Vasseur A	• 2022	NCT04158362	Phase III	1057		Taxane plus bevacizumab		HR = 0.77 [0.63; 0.94]	median OS: 24.9 months [20.0; 28.8], HR = 0.84 [0.68; 1.04])	In HR+/HER2- and HR-/HER2-, the negative outcome linked to paclitaxel was not noted anymore with the inclusion of bevacizumab.
6.	15913946	Nicolini A	• 2005			30 controls and 26 studied	aged 69 and 80 years,	Tamoxifen letrozole plus Immunotherapy	ER+/HER2-	12 months the patients on clinical benefit were	The two subgroups showed a similar surviv	This study indicates that immunotherapy has a significant impact on metastatic breast cancer

						patients				57% in the controls while they were 100% in the study group. At 31 months no control showed any benefit while 62% of the studied patients still maintained a benefit. Successively, in this group the	al (92.3% and 83.3% at 60 months	that is dependent on endocrine factors.	

										clinical benefit progressively decreased reaching 13.6% at 70 months		

Supplementary Table S3. Combination therapies with Immunotherapy/Chemotherapy + Targeted therapy/Endocrine therapy included in the analysis.

**Immunotherapy/Chemotherapy + Targeted therapy/Endocrine therapy**												
**S.No.**	**PMID**	**First Author**	**Publication Year**	**NCT number**	**Trial Phase**	**No. of patients**	**Age (median, range)**	**Immunotherapy/Chemotherapy + Targeted therapy/Endocrine therapy**	**Hormone receptor status, n (%)**	**PFS/EFS**	**OS/ORR/OR Median; 95% CI interval**	**Outcome**
1.	37256976	Turner C Nicholas	2024	NCT04305496.	phase III	708	≥20 years	355 patients assigned to the capivasertib–fulvestrant group and 353 to the placebo–fulvestrant	HR+ HER2-	7.3 months in the capivasertib–fulvestrant group, as compared with 3.1 months in the placebo–fulvestrant group HR, 0.50; 95% CI, 0.38 to 0.65; P<0.001		Capivasertib-fulvestrant therapy significantly prolonged progression-free survival compared to fulvestrant alone in patients with hormone receptor-positive advanced

												breast cancer that progressed after aromatase inhibitor therapy, with or without a CDK4/6 inhibitor.
2.	23679192	Yamamoto Y,	2013	UMIN000001841.		TOR 120 (N = 46) and EXE (n = 45)	71.6	Exemestane versus anastrozole	HR+ HER2 -	The PFS of TOR120 was longer than that of EXE, the difference being statistically significant (HR, 0.61, P = 0.045)	OS (HR, 0.60; P = 0.22).	TOR120 may offer greater benefits than EXE as a follow-up endocrine therapy for mBC patients who did not respond to non-steroidal AI treatment.

3.	37348019	Tolaney SM	2023	NCT04059484	Phase II	290		Amcenestrant Versus Endocrine Monotherapy	HR+ HER2-	3.6 *v* 3.7 months; stratified HR [HR], 1.051 [95% CI, 0.789 to 1.4]; one-sided *P* = .643)		AMEERA-3 did not achieve improved PFS with amcenestrant, but a numerical benefit was seen in ESR1 mutation patients. Efficacy and safety aligned with standard care for ER+/HER2- aBC.
4.	38833643	Chavez-MacGregor	2024	NCT01674140.	phaseIII	1,792		Everolimus	HR+ HER2-	HR, 0.94 [95% CI, 0.77 to 1.14]	HR, 0.97 [95% CI, 0.75 to 1.26].	A year of adjuvant everolimus with endocrine therapy did not improve overall

												outcomes. Subset analysis suggests mTOR inhibition may benefit premenopausal patients post-chemotherapy.
5.	35605174	Bachelot T	2022	NCT01805271	phaseII	1278		Everolimus	HR+ HER2-	ET plus everolimus (88% [95% CI, 85 to 91]) or ET plus placebo (89% [95% CI, 86 to 91; HR, 0.95; 95% CI, 0.69 to 1.32; P = .77]).		In high-risk patients, adding everolimus to adjuvant endocrine therapy did not improve survival. Tolerability was an issue, with over half of patients

												stopping the treatment early. Thus, everolimus is not recommended in the adjuvant setting.
6.	34425406	Kahan Z	2021	NCT02028507	phaseIII	537		palbociclib plus endocrine therapy versus capecitabine	HR+ HER2-	8.3 months for palbociclib/ET versus 5.3 months for capecitabine (adjusted HR, 0.70; 95% CI, 0.55-0.89; P = 0.003)		Patients on palbociclib/ET experienced a notable delay in GHS/QoL deterioration and better tolerance compared to those on capecitabine.

7.	33385521	Martin M	2021			296 and 305	≥18	palbociclib + exemestane or capecitabine	HR+ HER2 -	7.5 versus 10.0 months; HR 0.67; 95% CI: 0.53-0.85		Palbociclib plus ET did not show better PFS than capecitabine in AI-resistant MBC patients but demonstrated a safer profile and improved quality of life.
8.	26092818	Hurvitz SA	2015	NCT00876395	Phase III	480+239	≥18	everolimus with trastuzumab plus paclitaxel	HR+ HER2 -	14·95 months (95% CI 14·55-17·91) with everolimus versus 14·49 months (12·29-17·08)		Safety was consistent with BOLERO-3, emphasizing the need for proactive monitoring of

										with placebo (HR 0·89, 95% CI 0·73-1·08; p=0·1166).		adverse events.

Supplementary Table S4. PRISMA 2020 Main Checklist

Topic	No.	Item	Location where item is reported
**TITLE**			
**Title**	1	Identify the report as a systematic review.	Page 1
**ABSTRACT**			
**Abstract**	2	See the PRISMA 2020 for Abstracts checklist	
**INTRODUCTION**			
**Rationale**	3	Describe the rationale for the review in the context of existing knowledge.	Page 2
**Objectives**	4	Provide an explicit statement of the objective(s) or question(s) the review addresses.	Page 3
**METHODS**			
**Eligibility criteria**	5	Specify the inclusion and exclusion criteria for the review and how studies were grouped for the syntheses.	Page 3
**Information sources**	6	Specify all databases, registers, websites, organisations, reference lists and other sources searched or consulted to identify studies. Specify the date when each source was last searched or consulted.	Page 3
**Search strategy**	7	Present the full search strategies for all databases, registers and websites, including any filters and limits used.	Page 3

Topic	No.	Item	Location where item is reported
**Selection process**	8	Specify the methods used to decide whether a study met the inclusion criteria of the review, including how many reviewers screened each record and each report retrieved, whether they worked independently, and if applicable, details of automation tools used in the process.	Page 3 and 4
**Data collection process**	9	Specify the methods used to collect data from reports, including how many reviewers collected data from each report, whether they worked independently, any processes for obtaining or confirming data from study investigators, and if applicable, details of automation tools used in the process.	Page 3 and 4
**Data items**	10a	List and define all outcomes for which data were sought. Specify whether all results that were compatible with each outcome domain in each study were sought (e.g. for all measures, time points, analyses), and if not, the methods used to decide which results to collect.	Page 3 and 4; Table 1
	10b	List and define all other variables for which data were sought (e.g. participant and intervention characteristics, funding sources). Describe any assumptions made about any missing or unclear information.	Page 5 to 21; Table 1
**Study risk of bias assessment**	11	Specify the methods used to assess risk of bias in the included studies, including details of the tool(s) used, how many reviewers assessed each study and whether they worked independently, and if applicable, details of automation tools used in the process.	Page 4
**Effect measures**	12	Specify for each outcome the effect measure(s) (e.g. risk ratio, mean difference) used in the synthesis or presentation of results.	Page 3

Topic	No.	Item	Location where item is reported
**Synthesis methods**	13a	Describe the processes used to decide which studies were eligible for each synthesis (e.g. tabulating the study intervention characteristics and comparing against the planned groups for each synthesis (item 5)).	Page 3 and 4
	13b	Describe any methods required to prepare the data for presentation or synthesis, such as handling of missing summary statistics, or data conversions.	Page 3 and 4
	13c	Describe any methods used to tabulate or visually display results of individual studies and syntheses.	Table 1
	13d	Describe any methods used to synthesize results and provide a rationale for the choice(s). If meta-analysis was performed, describe the model(s), method(s) to identify the presence and extent of statistical heterogeneity, and software package(s) used.	Page 3 and 4
	13e	Describe any methods used to explore possible causes of heterogeneity among study results (e.g. subgroup analysis, meta-regression).	N/A
	13f	Describe any sensitivity analyses conducted to assess robustness of the synthesized results.	N/A
**Reporting bias assessment**	14	Describe any methods used to assess risk of bias due to missing results in a synthesis (arising from reporting biases).	Page 4
**Certainty assessment**	15	Describe any methods used to assess certainty (or confidence) in the body of evidence for an outcome.	Page 3 and 4
**RESULTS**			

Topic	No.	Item	Location where item is reported
**Study selection**	16a	Describe the results of the search and selection process, from the number of records identified in the search to the number of studies included in the review, ideally using a flow diagram.	Page 4
	16b	Cite studies that might appear to meet the inclusion criteria, but which were excluded, and explain why they were excluded.	Page 4 and 5; PRISMA flow chart
**Study characteristics**	17	Cite each included study and present its characteristics.	Table 1
**Risk of bias in studies**	18	Present assessments of risk of bias for each included study.	Page 19
**Results of individual studies**	19	For all outcomes, present, for each study: (a) summary statistics for each group (where appropriate) and (b) an effect estimate and its precision (e.g. confidence/credible interval), ideally using structured tables or plots.	Page 16-18, Reslut Section
**Results of syntheses**	20a	For each synthesis, briefly summarise the characteristics and risk of bias among contributing studies.	Page 16-19
	20b	Present results of all statistical syntheses conducted. If meta-analysis was done, present for each the summary estimate and its precision (e.g. confidence/credible interval) and measures of statistical heterogeneity. If comparing groups, describe the direction of the effect.	Page 16-19
	20c	Present results of all investigations of possible causes of heterogeneity among study results.	N/A

Topic	No.	Item	Location where item is reported
	20d	Present results of all sensitivity analyses conducted to assess the robustness of the synthesized results.	N/A
**Reporting biases**	21	Present assessments of risk of bias due to missing results (arising from reporting biases) for each synthesis assessed.	N/A
**Certainty of evidence**	22	Present assessments of certainty (or confidence) in the body of evidence for each outcome assessed.	Page 16-19
**DISCUSSION**			
**Discussion**	23a	Provide a general interpretation of the results in the context of other evidence.	Page 19
	23b	Discuss any limitations of the evidence included in the review.	Page 20
	23c	Discuss any limitations of the review processes used.	Page 20
	23d	Discuss implications of the results for practice, policy, and future research.	Page 21
**OTHER INFORMATION**			
**Registration and protocol**	24a	Provide registration information for the review, including register name and registration number, or state that the review was not registered.	Used PRISMA guidelines
	24b	Indicate where the review protocol can be accessed, or state that a protocol was not prepared.	N/A

Topic	No.	Item	Location where item is reported
	24c	Describe and explain any amendments to information provided at registration or in the protocol.	N/A
**Support**	25	Describe sources of financial or non-financial support for the review, and the role of the funders or sponsors in the review.	Page 21
**Competing interests**	26	Declare any competing interests of review authors.	Page 21
**Availability of data, code and other materials**	27	Report which of the following are publicly available and where they can be found: template data collection forms; data extracted from included studies; data used for all analyses; analytic code; any other materials used in the review.	Table 1

Supplementary Table S5. PRISMA Abstract Checklist

Topic	No.	Item	Reported?
**TITLE**			
**Title**	1	Identify the report as a systematic review.	Yes
**BACKGROUND**			
**Objectives**	2	Provide an explicit statement of the main objective(s) or question(s) the review addresses.	Yes
**METHODS**			
**Eligibility criteria**	3	Specify the inclusion and exclusion criteria for the review.	No
**Information sources**	4	Specify the information sources (e.g. databases, registers) used to identify studies and the date when each was last searched.	No
**Risk of bias**	5	Specify the methods used to assess risk of bias in the included studies.	No
**Synthesis of results**	6	Specify the methods used to present and synthesize results.	Yes
**RESULTS**			
**Included studies**	7	Give the total number of included studies and participants and summarise relevant characteristics of studies.	Yes
**Synthesis of results**	8	Present results for main outcomes, preferably indicating the number of included studies and participants for each. If meta-analysis was done, report the summary estimate and confidence/credible interval. If comparing groups, indicate the direction of the effect (i.e. which group is favoured).	No

Topic	No.	Item	Reported?
**DISCUSSION**			
**Limitations of evidence**	9	Provide a brief summary of the limitations of the evidence included in the review (e.g. study risk of bias, inconsistency and imprecision).	No
**Interpretation**	10	Provide a general interpretation of the results and important implications.	Yes
**OTHER**			
**Funding**	11	Specify the primary source of funding for the review.	No
**Registration**	12	Provide the register name and registration number.	No

From: Page, M.J.; McKenzie, J.E.; Bossuyt, P.M.; Boutron, I.; Hoffmann, T.C.; Mulrow, C.D.; Shamseer, L.; Tetzlaff, J.M.; Akl, E.A.; Brennan, S.E.; et al. The PRISMA 2020 statement: an updated guideline for reporting systematic reviews. *BMJ* **2021**, *372*, n71. https://doi.org/10.31222/osf.io/v7gm2.
